# Massive vulvar edema during pregnancy: A case report

**DOI:** 10.1016/j.ijscr.2022.107674

**Published:** 2022-09-20

**Authors:** Hamza Kiram, Maryem Bouab, Mohamed Jalal, Amine Lamrissi, Said Bouhya

**Affiliations:** Obstetrics And Gynecology Department, University Hospital Center Ibn Rochd, Casablanca, Morocco; Faculty of Medicine and Pharmacy, Hassan II University, Casablanca, Morocco

**Keywords:** Vulvar edema, Pregnancy, Preeclampsia, Case report

## Abstract

**Introduction:**

Massive vulvar edema is an unusual complication of pregnancy that may be due to underlying systemic pathology but has also been associated with preeclampsia. It is likely to interfere with vaginal delivery. It has been associated with maternal mortality in the postpartum period.

**Observation:**

A 34 years old female patient, having already delivered a child by vaginal route, without any other particular pathological history, admitted for severe pre-eclampsia on an unattended pregnancy estimated at 36 weeks of amenorrhea.

The examination on admission showed a blood pressure of 170/110 mmHg, a proteinuria of three crosses on the urine dipstick. Examination of the vulva showed massive vulvar edema. The massive vulvar edema was explained by hypoprotidemia secondary to renal damage in the context of severe pre-eclampsia.

The evolution was marked by the rapid normalization of the tentional figures and a spectacular regression of the vulvar edema, and its complete disappearance in fifteen days.

**Discussion:**

Edema may be seen in 80 % of pregnant women, but isolated massive vulvar edema is rare in pregnancy. Massive vulvar edema has been reported in the literature after tocolysis, vulvovaginitis, Crohn's disease and pre-eclampsia.

Treatment of vulvar edema is necessary because it can be alarming to the patient and may lead to occlusion of the vulvar orifices.

The patient with vulvar edema deserves special attention, and identification and treatment of the associated factors are essential to its management.

**Conclusion:**

Massive vulvar edema is rare in pregnancy but requires special attention because it can be associated with maternal and fetal complications.

Treatment is symptomatic and etiologic whenever an underlying cause is found and the evolution is often favorable with proper treatment.

## Highlights

• Massive vulvar edema is an unusual complication of pregnancy
• It may be due to underlying systemic pathology but has also been associated with preeclampsia.
• Treatment is symptomatic and etiologic whenever an underlying cause is found and the evolution is often favorable with proper treatment.

## 1 Introduction

Massive vulvar edema is an unusual complication of pregnancy that may be due to underlying systemic pathology but has also been associated with preeclampsia. It is likely to interfere with vaginal delivery. It has been associated with maternal mortality in the postpartum period [1].

We present the case of a patient with massive vulvar edema related to her preeclampsia in a 36-weeks of amenorrhea pregnancy with dramatic disappearance in the postpartum period.

This work has been reported with respect to the SCARE 2020 criteria [2].

## 2 Observation

**Fig. 1:**
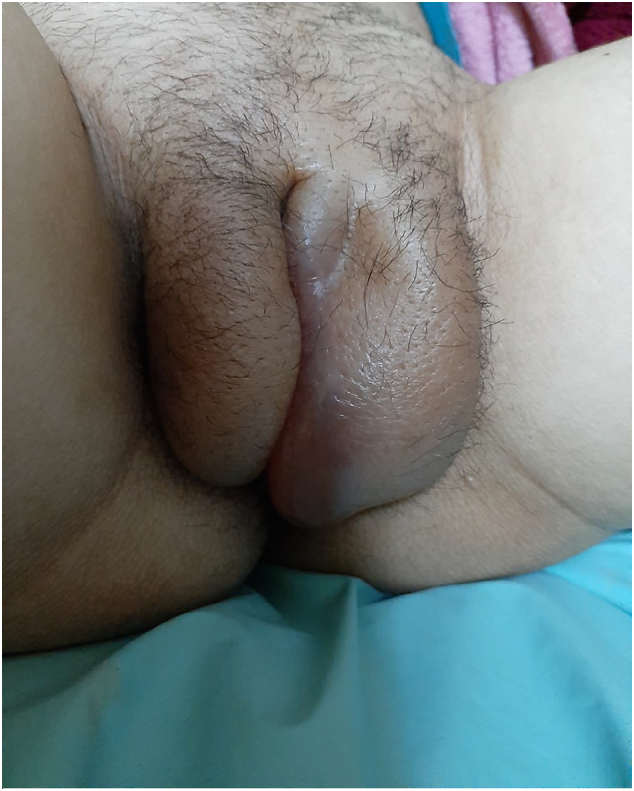
Significant vulvar edema predominantly on the left

A 34 years old female patient, second gesture, having already delivered a child by vaginal route, without any other particular pathological history, admitted for severe pre-eclampsia on an unattended pregnancy estimated at 36 weeks of amenorrhea with a significant vulvar edema of rapid evolution (Fig. 1).

The patient was apyretic, on examination, there was no evidence of vulvar trauma, infection or medication. The examination on admission showed a blood pressure of 170/110 mmHg, a proteinuria of three crosses on the urine dipstick. Examination of the vulva showed massive vulvar edema involving the labia minora and majora, predominantly on the left, with fissuring of the inner side of the right majora, with a rocky liquid discharge. The obstetrical examination showed a tense uterus with a closed cervix and an intact water sac, the presentation was cephalic. Fetal heart rate recording showed a flat pattern without accelerations. The rest of the examination showed no signs of thrombosis or regional adenopathy apart from minor edema of the lower limbs. A cesarean section was performed for suspected retroplacental hematoma. It allowed the extraction of a female newborn 2500 g, Apgar 10/10, entrusted to the pediatrician.

The biological examination on admission showed a mild microcytic hypochromic anemia (hemoglobin 10 g/dL), a normal platelet count, a normal prothrombin level and transaminases, a negative infectious workup, hypoprotidemia at 21 g/L, and 24-h proteinuria at 4 g/24 h. The massive vulvar edema was explained by hypoprotidemia secondary to renal damage in the context of severe pre-eclmaplsia. The treatment consisted of local care with magnesium sulphate associated with an increase in daily protein intake in addition to the termination of the pregnancy by cesarean section, which treated the etiology and promoted venous return.

The evolution was marked by the rapid normalization of the tentional figures and a spectacular regression of the vulvar edema, and its complete disappearance in fifteen days.

## 3 Discussion

Edema may be seen in 80 % of pregnant women [3], but isolated massive vulvar edema is rare in pregnancy.

Massive vulvar edema has been reported in the literature after tocolysis, vulvovaginitis, Crohn's disease and pre-eclampsia [4], [5], [6].

.Other possible causes of vulvar edema are local trauma, infections and vascular and lymphatic obstructions. None of these could be observed in our patient.

We have found only a few cases of idiopathic postpartum vulvar edema [7].

The differential diagnosis of vulvar edema includes infections, tumors, congenital lymphatic anomalies, trauma, inflammatory diseases, and metabolic diseases. Interestingly, vulvar edema occurring in the immediate postpartum period has been reported with maternal deaths due to vascular collapse [8].

The development of edema during a normal pregnancy is multifactorial. It involves changes in estrogen levels, activation of the renin-angiotensin system, and compression of the inferior vena cava by uterine volume.

In pre-eclampsia, the increase in capillary pressure and the decrease in oncotic pressure due to hypo-albuminemia bring water back into the interstitial environment [9], [10].

.The vulvar edema in our patient was probably due to hypoprotidemia which is quite often associated with severe pre-eclampsia. The topical application of magnesium sulfate dressings would help to decrease in venous perfusion pressure in the vulval areolar of the richly vascularized vulvar areolar tissue.

Treatment of vulvar edema is necessary because it can be alarming to the patient and may lead to occlusion of the vulvar orifices.

The patient with vulvar edema deserves special attention, and identification and treatment of the associated factors are essential to its management [11].

## 4 Conclusion

Massive vulvar edema is rare in pregnancy but requires special attention because it can be associated with maternal and fetal complications. Its association with pre-eclampsia has only been reported in the literature in a few cases.

Treatment is symptomatic and etiologic whenever an underlying cause is found and the evolution is often favorable with proper treatment.

## Provenance and peer review

Not commissioned, externally peer-reviewed.

## Consent

Written informed consent for publication of their clinical details and/or clinical images was obtained from the patient.

## Ethical approval

I declare on my honor that the ethical approval has been exempted by my establishment.

## Sources of funding

None.

## Guarantor

B.M

## Registration of research studies

None.

## CRediT authorship contribution statement

All the authors contributed in data collection, manuscript drafting and reviewing, and approval of final manuscript.

## Declaration of competing interest

The authors declare having no conflicts of interest for this article.
